# Expression of glutathione S-transferase B1, B2, Mu and Pi in breast cancers and their relationship to oestrogen receptor status.

**DOI:** 10.1038/bjc.1989.375

**Published:** 1989-12

**Authors:** A. F. Howie, W. R. Miller, R. A. Hawkins, A. R. Hutchinson, G. J. Beckett

**Affiliations:** University Department of Clinical Chemistry, Royal Infirmary, Edinburgh, UK.

## Abstract

The concentrations of glutathione S-transferase (GST) B1 and B2 (Alpha), Pi and Mu have been measured by radioimmunoassay in cytosols from 28 oestrogen receptor (ER) rich an 30 ER-poor breast tumours. GST B1, B2 and Pi was detected in all 58 breast tumour cytosols whilst GST Mu was found in only 28. Of the GSTs, Pi was expressed most strongly in all cytosols and the concentration was significantly higher in ER-poor tumour cytosols than in ER-rich tumours (P less than 0.01). As with GST Pi, the highest levels of GST B1 and GST B2 were found in ER-poor tumour cytosols; the levels of GST B1 and GST B2 were positively correlated (r = 0.66, P less than 0.001). No quantitative or qualitative association was found between ER status and GST Mu which was expressed in 46% of ER-rich and 50% of ER-poor tumour cytosols. No relationship could be found between GST expression and age, menopausal status, lymph node involvement or tumour T stage in the subgroup of patients in whom this information was available. These data suggest that a common mechanism is responsible for GST induction in ER-poor tumours and that the nulled Mu phenotype has no increased susceptibility to developing breast cancer.


 © The Macmillan Press Ltd., 1989

### Expression of glutatione S-transferase $B_1$, $B_2$, Mu and Pi in breast cancers and their relationship to oestrogen receptor status

A.F. Howie[1], W.R. Miller[2], R.A. Hawkins[2], A.R. Hutchinson[1] & G.J. Beckett[1]

*University Departments of [1]Clinical Chemistry and [2]Surgery, The Royal Infirmary, Edinburgh EH3 9YW, UK.*

**Summary** The concentrations of glutathione S-transferase (GST) $B_1$ and $B_2$ (Alpha), Pi and Mu have been measured by radioimmunoassay in cytosols from 28 oestrogen receptor (ER) rich and 30 ER-poor breast tumours. GST $B_1$, $B_2$ and Pi was detected in all 58 breast tumour cytosols whilst GST Mu was found in only 28. Of the GSTs, Pi was expressed most strongly in all cytosols and the concentration was significantly higher in ER-poor tumour cytosols than in ER-rich tumours ($P<0.01$). As with GST Pi, the highest levels of GST $B_1$ and GST $B_2$ were found in ER-poor tumour cytosols; the levels of GST $B_1$ and GST $B_2$ were positively correlated ($r=0.66$, $P<0.001$). No quantitative or qualitative association was found between ER status and GST Mu which was expressed in 46% of ER-rich and 50% of ER-poor tumour cytosols. No relationship could be found between GST expression and age, menopausal status, lymph node involvement or tumour T stage in the subgroup of patients in whom this information was available. These data suggest that a common mechanism is responsible for GST induction in ER-poor tumours and that the nulled Mu phenotype has no increased susceptibility to developing breast cancer.

The glutathione S-transferases (GST) are a family of dimeric enzymes which are found in the cell cytoplasm. They have been implicated in the detoxification of a wide range of xenobiotics and chemotherapeutic agents (Jakoby, 1978; Mannervik, 1985; Buller *et al.*, 1987). Three immunologically distinct major classes of GST occur in humans Alpha (that comprises $B_1$ and $B_2$ subunits), Mu and Pi (Mannervik *et al.*, 1985; Stockman *et al.*, 1985). Although present in most human cells, the expression of the various GST isoenzymes may vary between different types of tissue (Strange *et al.*, 1984). Approximately half the population do not express GST Mu and appear more susceptible to developing lung cancer if they smoke heavily (Seidegard *et al.*, 1986). GST Pi has been associated with pre-neoplastic and neoplastic change and may be present in many human tumours and cell lines (Ilio *et al.*, 1986; Kodate *et al.*, 1986; Shiratori *et al.*, 1987; Shea *et al.*, 1988; Mannervik *et al.*, 1987).

Using a cDNA probe, over-expression of GST Pi RNA has been found in a multidrug-resistant cell line of breast cancer and also in a small series of 21 breast cancers (Moscow *et al.*, 1988). In the same study an inverse correlation was found between expression of GST Pi and the oestrogen receptor levels in the tumour. The expression of the Alpha and Mu classes of GST was not measured.

In the present study we have used specific radioimmunoassays to measure the expression, at the protein level, of GST Pi, Mu, $B_1$ and $B_2$ in cytosols from a larger group of 58 breast cancers.

### Methods

### *Patients*

Fifty-eight women with histologically proven invasive breast cancer were studied. Apart from four patients (three treated with tamoxifen and one with chemotherapy), none had received prior therapy for their malignancy.

Tumour was obtained from the primary cancer in 53 cases and from an axillary lymph node invaded with cancer in the remaining five individuals. The tumours were transported on ice to a cold room and then stored in liquid nitrogen until assayed.

### *Cytosol preparation*

All procedures were performed at 0–4°C. Tumour was dissected from surrounding fat and connective tissue, finely cut with scissors and homogenised in 20 mmol $l^{-1}$ Tris buffer, pH 7.5 (w/v 1:10), using a Silverson homogeniser at maximum speed for 20 s, then 15 with 1 min interval for cooling. The homogenate was then centrifuged at 105,000 *g* for 1 h and the resultant supernatant was used as a cytosol.

### *Oestrogen receptors*

Oestrogen receptors were measured in an adjacent portion of tumour by saturation analysis (Hawkins *et al.*, 1981). Tumour cytosol was incubated overnight at 4°C with $^3$H-17 β-oestradiol. Separation of free and bound steroid was by addition of dextran-coated charcoal; the bound fraction was measured by liquid scintillation counting. Concentration of receptors was determined by Scatchard analysis (Scatchard, 1949). Activities below 20 pmol $mg^{-1}$ cytosol protein were designated oestrogen receptor poor (ER-poor) and those with activities in excess of 20 pmol $mg^{-1}$ cytosol protein as ER-rich.

### *Measurement of GST concentrations*

Specific radioimmunoassays, described in detail previously (Beckett & Hayes, 1984; Hussey *et al.*, 1987; Howie *et al.*, 1988), were used to determine the concentration of GST Pi, GST Mu, GST $B_1$ and GST $B_2$ isoenzymes in the 58 breast cytosols. The standards used for the $B_1$, $B_2$ and Mu assays were purified from human liver obtained from transplant donors and for GST Pi from fresh human placenta.

The cytosol, if required, was diluted in assay diluent consisting of 25 mmol $l^{-1}$ sodium phosphate buffer pH 7.6 bovine serum albumin (1 g $l^{-1}$) and sodium azide (0.2 g $l^{-1}$) before analysis. Within-assay coefficients of variation of less than 10% was achieved in all assays.

### *Protein measurement*

The dye binding technique described by Bradford (1976), adapted for use on a Cobas Fara (Roche Diagnostic Ltd, Welwyn Garden City, UK) centrifugal analyser was used for total protein estimation.

### Results

The median levels of each of the GST isoenzymes in the breast cytosols are shown in Table I. GST Pi, $B_1$ and $B_2$ were detected in all breast tumour cytosols, whereas GST Mu was found in only 28 (48%) of the 58 cytosols. GST Pi was expressed most strongly with concentrations ranging from 30 to 1,110 $\mu g.g^{-1}$ cytosolic protein. For GST $B_1$ and $B_2$ the

Correspondence: G.J. Beckett.
Received 4 May 1989; and in revised form 21 July 1989.

**Table I** Median GST levels

| | *Median GST concentrations (μg GST g⁻¹ protein)* | | | |
|---|---|---|---|---|
| | $B_1$ | $B_2$ | *Pi* | *Mu* |
| All tumours (*n* = 58) | 4.5 | 0.86 | 222 | 118 |
| ER-rich tumours (*n* = 28) | 3.6 | 0.98 | 198 | 183 |
| ER-poor tumours (*n* = 30) | 4.7 | 0.82 | 306 | 107 |

For GST Mu, data is given for only the 28 tumours that expressed the protein.

concentrations ranged from 0.6 to 48 $\mu g\ g^{-1}$ cytosolic protein and 0.04 to 5.3 $\mu g\ g^{-1}$ cytosolic protein, respectively (Figure 1). The levels of $B_1$ and $B_2$ were positively correlated ($r = 0.66$, $P<0.001$) but GST Pi did not correlate with the expression of other GST.

The concentrations of GST in the cytosols subdivided according to the ER status as shown in Figure 1. Levels of GST Pi were significantly higher in ER-poor tumours ($P<0.01$, Mann–Whitney U test). Indeed, the tumours with the 11 highest GST Pi values were in the ER-poor tumours. As with GST Pi, the highest levels of GST $B_1$ and GST $B_2$ were observed in ER-poor tumours with the eight highest GST $B_1$ values being found in the ER-poor tumour group. However, when compared as groups the differences in GST $B_1$ and GST $B_2$ between ER-rich and ER-poor tumours did not reach statistical significance. No significant correlation could be found in the quantitative levels of oestrogen receptors and the expression of any of the GST classes.

No association was found between GST Mu and ER status. GST Mu was present in 15 of the 30 ER-poor tumours and 13 of the 28 ER-rich tumours. The same pattern of GST expression between groups was observed if a level of $<5$ pmol $mg^{-1}$ was used to define ER status.

There was no relationship between levels of GSTs and the age or menopausal status of the patients from whom the tumour was derived. Furthermore, no obvious association was apparent between GST and lymph node involvement or tumour T stage in the subgroup of patients in whom this information was available. Similarly, there was no indication that tumour cellularity, central necrosis or a special histological type was associated with increased expression of GSTs.

The frequency with which GST Mu was expressed in the breast cancers was not significantly different from the frequency of expression of GST Mu found using lymphocytes obtained from a group of 42 laboratory volunteers where 23 (55%) volunteers expressed the enzyme (test of confidence limits for a proportion; Snedecor & Cochran, 1974).

### Discussion

Our results concerning the elevated expression of GST Pi in ER-poor breast cancer are compatible with the data of Moscow *et al.* (1988), who showed increased mRNA levels for GST Pi in a similar group of tumours. This suggests that raised mRNA levels in ER-poor tumours results in an increased production of GST Pi protein. In addition, we have shown that both $B_1$ and $B_2$ GST appear to be over-expressed in some ER-poor tumours.

Although the role of GST Pi in conferring drug resistance has been recently questioned (Yusa *et al.*, 1988) an association between GST Pi expression and acquisition of drug resistance has been widely reported (for review see Hayes & Wolf, 1988). It has been suggested that the increased levels of GST Pi in ER-poor tumours will confer a poorer prognosis because such patients will have a lesser chance of responding to chemotherapeutic agents (Moscow *et al.*, 1988). However,

**Figure 1** Concentrations of GST class Alpha ($B_1$ and $B_2$) and Pi and mu in oestrogen receptor rich (ER+) and poor (ER−) breast tumours (●). Tumours in which the concentration of GST Pi exceeded 400 $\mu g\ g^{-1}$ cytosolic protein are shown by O.

it should be noted that ER-poor tumours often have a high proliferation rate (Meyer *et al.*, 1977) and this can be associated with rapid response to chemotherapy (Stoll, 1986). Additionally, there is no consensus on whether response rates to chemotherapy differ significantly in ER-rich and ER-poor tumours (Hawkins *et al.*, 1980; Carle *et al.*, 1984). In rat mammary tumours increases in the Alpha class GST are also associated with resistance to chemotherapeutic agents (Buller *et al.*, 1987). In our study 11 ER-poor tumours over-expressed GST Pi while six and four ER-poor tumour over-expressed GST $B_1$ and GST $B_2$, respectively. However, we have no clinical evidence that these increased Alpha class and Pi class GST levels are associated with chemotherapeutic insensitivity.

Aquisition of multi drug resistance in MCF-7 breast cancer cells is associated not only with an increase in GST Pi expression but also with a loss of hormone sensitivity. Our data show that both GST Pi and Alpha class GST expression is inversely linked to ER status. These observations are of interest since ER-poor tumours as a group are associated with worse prognosis than those which are ER-rich (McGuire *et al.*, 1975*a*; Nicolson *et al.*, 1981; Hawkins *et al.*, 1987; Courdi *et al.*, 1988). Whether this results from ER-rich cancers being inherently more indolent or more likely to respond to steroid hormone deprivation therapy is not clear (Howell *et al.*, 1984; Saez *et al.*, 1983; Adami *et al.*, 1985). However, it also has to be emphasised that whereas ER-poor tumours rarely respond to hormone manipulation (McGuire *et al.*, 1975*b*; Le Clerq & Heuson, 1977), the present results showed that only a small proportion of ER-poor tumours have elevated levels of GST. In this respect the observation that ER-negative breast cancers can be divided into sub-groups on the basis of other tumour characteristics such as epidermal growth factor receptors, vimentin and p53 expression is relevant (Cattoretti *et al.*, 1988). In the case of EGF receptors, this may be of prognostic value (Sainsbury *et al.*, 1987) but it is not known if there is an association between GST Pi expression and EGF receptor levels. It will be of interest to know whether patients having tumour with over-expression of GST will return more quickly with recurrent disease than those with lower GST, irrespective of ER status.

GST Pi maps to chromosome 11 (Moscow *et al.*, 1988), which has an increased incidence of deletions in ER-poor tumour cells when compared to ER-rich tumours (Ali *et al.*, 1987). These observations have led to the suggestion of a link between over-expression of GST Pi and a specific deletion on chromosome 11. The Alpha class of GST, however, map to chromosome 6 (Board & Webb, 1987) and we have shown that both GST $B_1$ and GST $B_2$ are also over-expressed in some ER-poor tumours. These data suggest that there may be a common mechanism, rather than specific chromosomal deletions, that leads to over-expression of the GST in certain breast cancers.

The incidence of GST Mu expression in both the ER-rich and ER-poor breast tumours was not significantly different from the incidence of GST Mu expression found in lymphocytes from the normal population (Seidegard *et al.*, 1986; Hussey *et al.*, 1987). This suggests a lack of association between expression of GST Mu and breast cancer.

In summary, glutathione S-transferases $B_1$, $B_2$ and Pi have been found in all cytosols of breast cancer whereas Mu was detected in only about one-half. High levels of GST $B_1$, $B_2$ and Pi were limited to ER-poor tumours, the clinical significance of which merits further study.

This work was supported by a grant from the Sir Stanley and Lady Davidson Medical Research Fund. A.R.H. was in receipt of a Scottish Home and Health Department Summer Vacation Studentship Grant.

### References

ADAMI, H.O., LINDGREN, A. & SALLSTROM, J. (1985). Prognostic implication of estrogen receptor content in breast cancer. *Breast cancer Res. Treat.*, **5**, 293.

ALI, I.U., LIDEREAU, R., THEILLET, C. & CALLAHAN, R. (1987). Reduction of homozygosity of genes on chromosome 11 in human breast neoplasia. *Science*, **238**, 185.

BECKETT, G.J. & HAYES, J.D. (1984). Development of specific radioimunoassays for the meaurement of human hepatic basic and N/A2b glutathione S-transferases. *Clin. Chim. Acta*, **141**, 267.

BOARD, P.G. & WEBB, G.C. (1987). Isolation of a cDNA clone and localization of human glutathione S-transferase 2 genes to chromosome band 6p 12. *Proc. Natl Acad. Sci. USA*, **84**, 2377.

BRADFORD, M. (1976). A rapid and sensitive method for the quantitation of microgram quantities of protein using the principle of protein-dye binding. *Anal. Biochem.*, **72**, 248.

BULLER, A.L., CLAPPER, M.L. & TEW, K.D. (1987) Glutathione S-transferases in nitrogen mustard resistant and sensitive cell lines. *Mol. Pharmacol.*, **31**, 575.

CARLE, B.K., SEARS, M.E. & OLSON, K.B. (1984). Relationship of quantitative estrogen receptor levels and clinical response to cytotoxic chemotherapy in advanced breast cancer. *Cancer*, **54**, 1554.

CATTORETTI, G., ANDREALA, S., CLEMENTE,C., D'AMATO, L. & RIKKE, F. (1988). Vimentin and p53 expression on epidermal growth factor receptor-positive, estrogen receptor-negative breast carcinomas. *Br. J. Cancer*, **57**, 353.

COURDI, A., HERY, M., CHAUVEL, P., GIOANNI, J., NAMER, M. & DEMARD, F. (1988). Prognostic value of continuous variables in breast cancer and head and neck cancer. Dependence on the cut-off level. *Br. J. Cancer*, **58**, 88.

HAWKINS, R.A., ROBERTS, M.M. & FORREST, A.P.M. (1980). Oestrogen receptors and breast cancer: current status. *Br. J. Surg.*, **67**, 153.

HAWKINS, R.A., BLACK, R., STEELE, R.T.C., DIXON, J.M. & FORREST, A.P.M. (1981). Oestrogen receptor concentration in primary breast cancer and axillary node metastasis. *Breast Cancer Res. Treat.*, **1**, 245.

HAWKINS, R.A., WHITE, G, BUNDRED, N.J. *et al.* (1987). Prognostic significance of oestrogen and progestogen receptor activities in breast cancer. *Br. J. Surg.*, **74**, 1009.

HAYES, J.D. & WOLF, C.R. (1988). Role of glutathione transferase in drug resistance. In *Glutathione Conjugation – Its Mechanism and Biological Significance*, Sies, H. & Ketterer. B. (eds) p. 315. Academic Press: London.

HOWELL, A., BARNES, D.M., HARLAND, R.N.L. *et al.* (1984). Steroid hormone receptors and survival after first relapse in breast cancer. *Lancet*, **i**, 588.

HOWIE, A.F., HAYES, J.D. & BECKETT, G.J. (1988). Purification of acidic glutathione S-transferase from human lung, placenta and erythrocytes and the development of a specific radioimmunoassay for their measurement. *Clin. Chim. Acta*, **177**, 65.

HUSSEY, A.J., HAYES, J.D. & BECKETT, G.J. (1987). The polymorphic expression of neutral glutathione S-transferase in human mononuclear leucocytes as measured by specific radioimmunoassay. *Biochem. Pharmacol.* **36**, 4013.

ILIO, C.D., BOCCIO, G.D., MASSOUD, R. & FEDERICI, G. (1986). Glutathione transferase of human breast is closely related to transferase of human placenta and erythrocytes. *Biochem. Int.*, **13**, 263.

JAKOBY, W.B. (1978). The glutathione S-transferases: a group of multi-functional detoxification proteins. *Adv. Enzymol.*, **46**, 383.

KODATE, C., FUKUSHI, A., NARITA, T., KUDO, H., SOMA, Y. & SATO, K. (1986). Human placental form of glutathione S-transferases (GST-π) as a new immunohistochemical marker for human colonic carcinoma. *Jpn. J. Cancer Res. (Gann)*, **77**, 226.

LE CLERQ, G. & HEUSON, J.C. (1977). Therapeutic significance of sex-steroid hormone receptors in the treatment of breast cancer. *Eur. J. Cancer*, **13**, 1205.

MCGUIRE, W.L., CARBONE, P.P., SEARS, M.E. & ESCHER, G.C. (1975*a*). Estrogen receptors in human breast cancer: an overview. In *Estrogen Receptors in Human Breast Cancer*, McGuire, W.L., Carbone, P.P. & Vollmer, E.P. (eds) p.1. Raven Press: New York.

MCGUIRE, W.L., PEARSON, O.H. & SEGALOFF, A. (1975*b*) Predicting hormone responsiveness in human breast cancers. In *Estrogen Receptors in Human Breast Cancer*, McGuire, W.L., Carbone, P.P. & Vollmer, E.P. (eds) p. 37. Raven Press: New York.

MANNERVIK, B. (1985). The isoenzymes of glutathione S-transferase. *Adv. Enzymol.*, **57**, 357.

MANNERVIK, B., ALIN, P., GUTHENBERG, C. *et al.* (1985). Identification of three classes of cytosolic glutathione transferase common to several mammalian species: correlation between structural data and enzymatic properties. *Proc. Natl Acad. Sci. USA*, **82**, 7202.

MANNERVIK, B., CASTRO, V.M., DANIELSON, U.H., TAHIR, M.K., HANSSON, J. & RINGBORG, U. (1987). Expression of class Pi glutathione transferase in human malignant melanoma cells. *Carcinogenesis*, **8**, 1929.

MEYER, J.S., RAO, B.R., STEVENS, S.C. & WHITE, W.L. (1977). Low incidence of estrogen receptor in breast carcinomas with rapid rates of cellular replication. *Cancer*, **40**, 2290.

MOSCOW, J.A., TOWNSEND, A.J., GOLDSMITH, M.E. *et al.* (1988). Isolation of the human anionic glutathione S-transferase cDNA and the relation of its gene expression to estrogen-receptor content in primary breast cancer. *Proc. Natl Acad. Sci. USA*, **85**, 6518.

NICHOLSON, R.I., CAMPBELL, F.C., BLAMEY, R.W., ELSTON, C.W., GEORGE, D. & GRIFFITHS, K. (1981). Steroid receptors in early breast cancer. *J. Steroid Biochem.*, **15**, 193.

SAEZ, S., CHEIX, F. & ASSELAIN, B. (1983). Prognostic value of estrogen and progesterone receptors in primary breast cancer. *Breast Cancer Res. Treat.*, **3**, 3345.

SAINSBURY, J.C., FARNDON, J.R., NEEDHAM, G.K., MALCOLM, A.J. & HARRIS, A.L. (1987). Epidermal-growth-factor receptor status as a predictor of early recurrence of and death from breast cancer. *Lancet*, **i**, 1398.

SCATCHARD, G. (1949). The attraction of protein on small molecules and ions. *Ann. NY Acad. Sci.*, **57**, 660.

SEIDEGARD, J., PERO, R.W., MILLER, D.G. & BEATTIE, E.J. (1986). A glutathione transferase in human leukocytes as a marker for the susceptibility to lung cancer. *Carcinogenesis*, **7**, 751.

SHEA, T.C., KELLEY, S.L. & HENNER, W.D. (1988). Identification of an anionic form of glutathione transferase present in many human tumours and human tumour cell lines. *Cancer Res.*, **48**, 527.

SHIRATORI, Y., SOMA, Y., MARUYAMA, H., SATO, S., TAKANO, A. & SATO, K. (1987). Immunohistochemical detection of the placental form of glutathione S-transferases in dysplastic and neoplastic human uterine cervix lesions *Cancer Res.*, **47**, 6806.

SNEDECOR, G.W. & COCHRAN, W.G. (1974). *Statistical Methods*, 6th edn, p.210. Iowa State University Press: Ames.

STOCKMAN, P.K., BECKETT, G.J. & HAYES, J.D. (1985). Identification of a basic hybrid glutathione S-transferase from human liver. Glutathione S-transferase $\delta$ is composed of two distinct subunits ($B_1$ and $B_2$). *Biochem. J.*, **227**, 457.

STOLL, B.A. (1986). Components of a prognostic index. In *Breast Cancer Treatment and Prognosis*, Stoll, B.A. (ed) p. 115. Blackwell Scientific: Oxford.

STRANGE, R.C., FAULDER, C.G., DAVIS, B.A. *et al.* (1984). The human glutathione S-transferases: studies on the tissue distribution and genetic variation of the GST 1, GST2 and GST3 isoenzymes. *Ann. Human Genet.*, **48**, 11.

YUSA, K., HAMADA, H. & TSURUO, T. (1988). Comparison of glutathione S-transferase activity between drug-resistant and -sensitive human tumour cells: is glutathione S-transferase associated with multi-drug resistance? *Cancer Chemother. Pharmacol.*, **22**, 17.


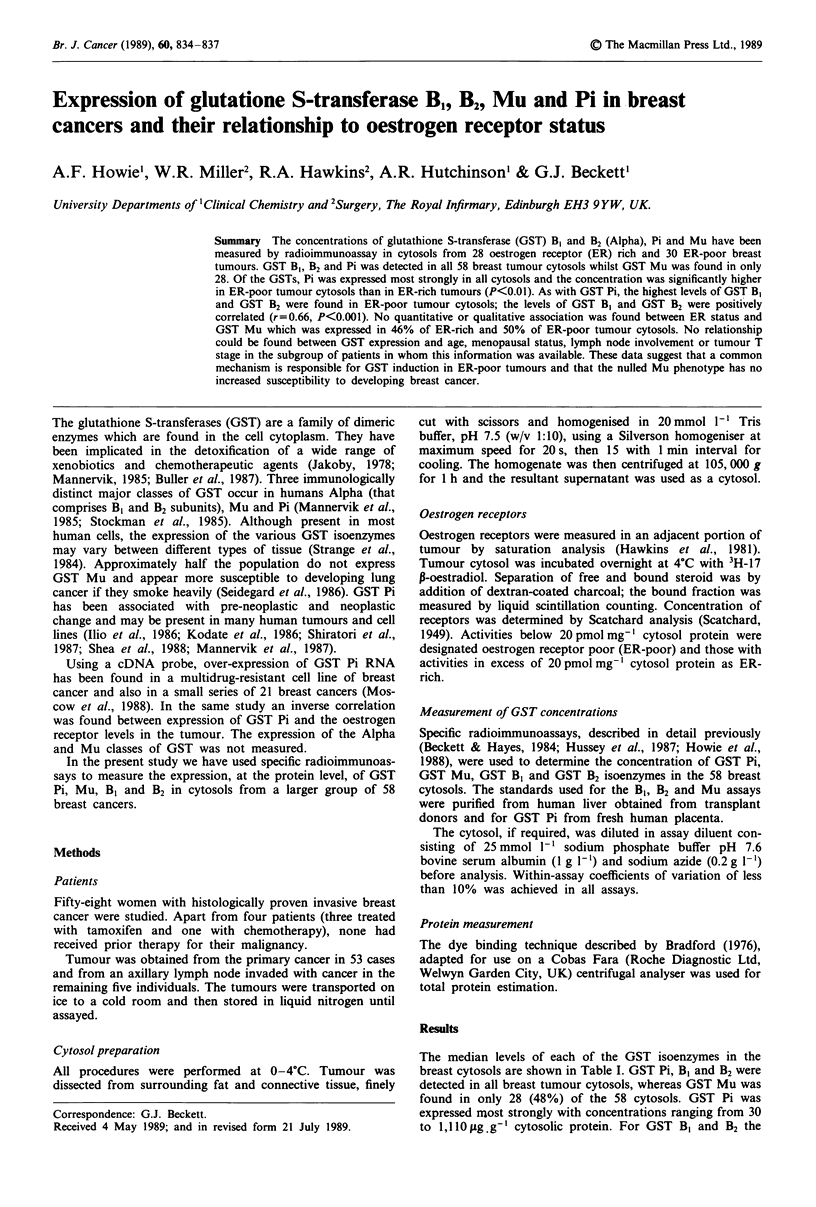

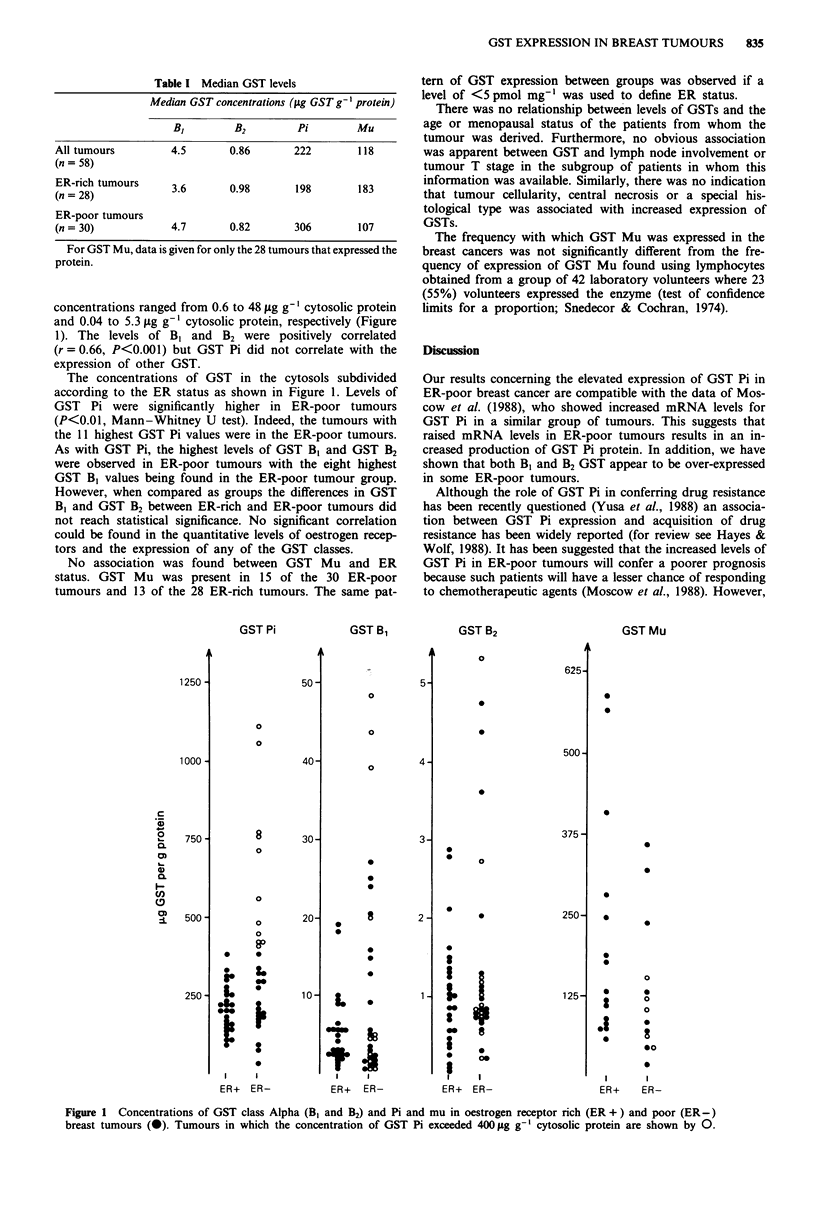

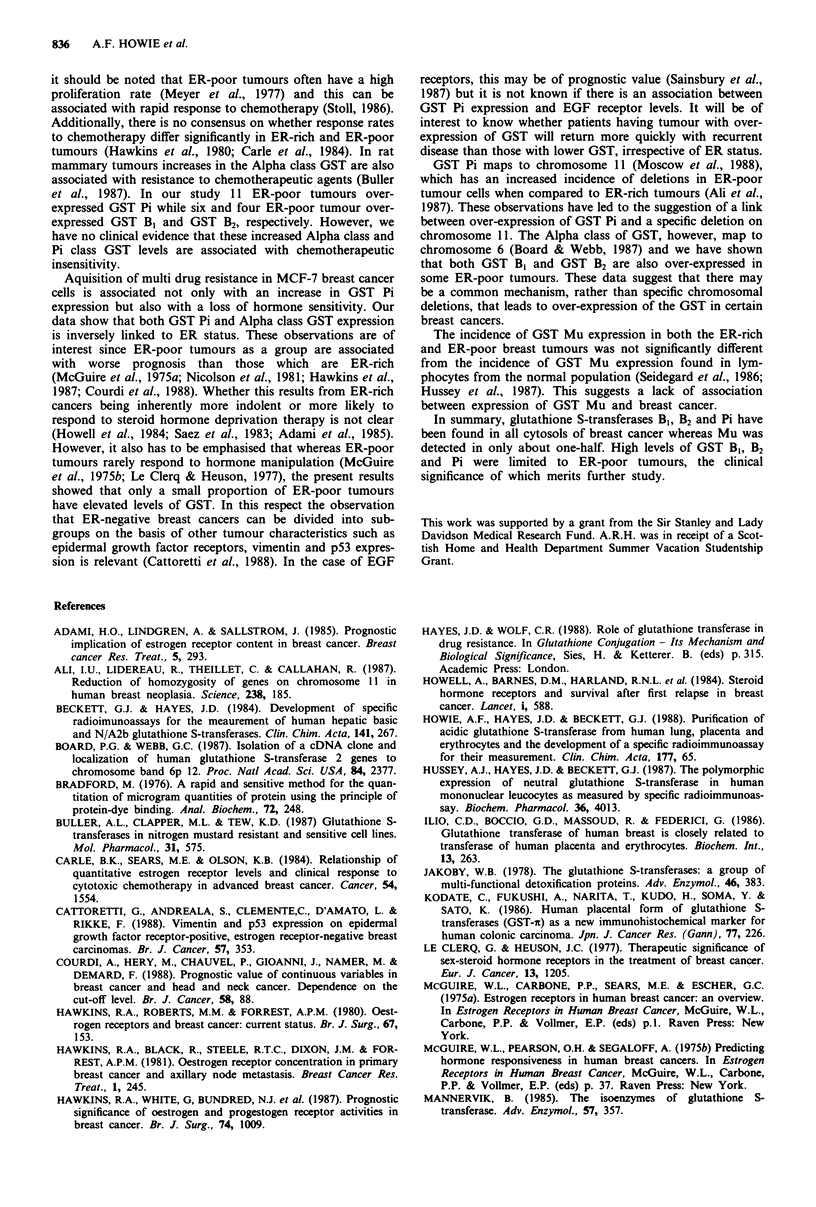

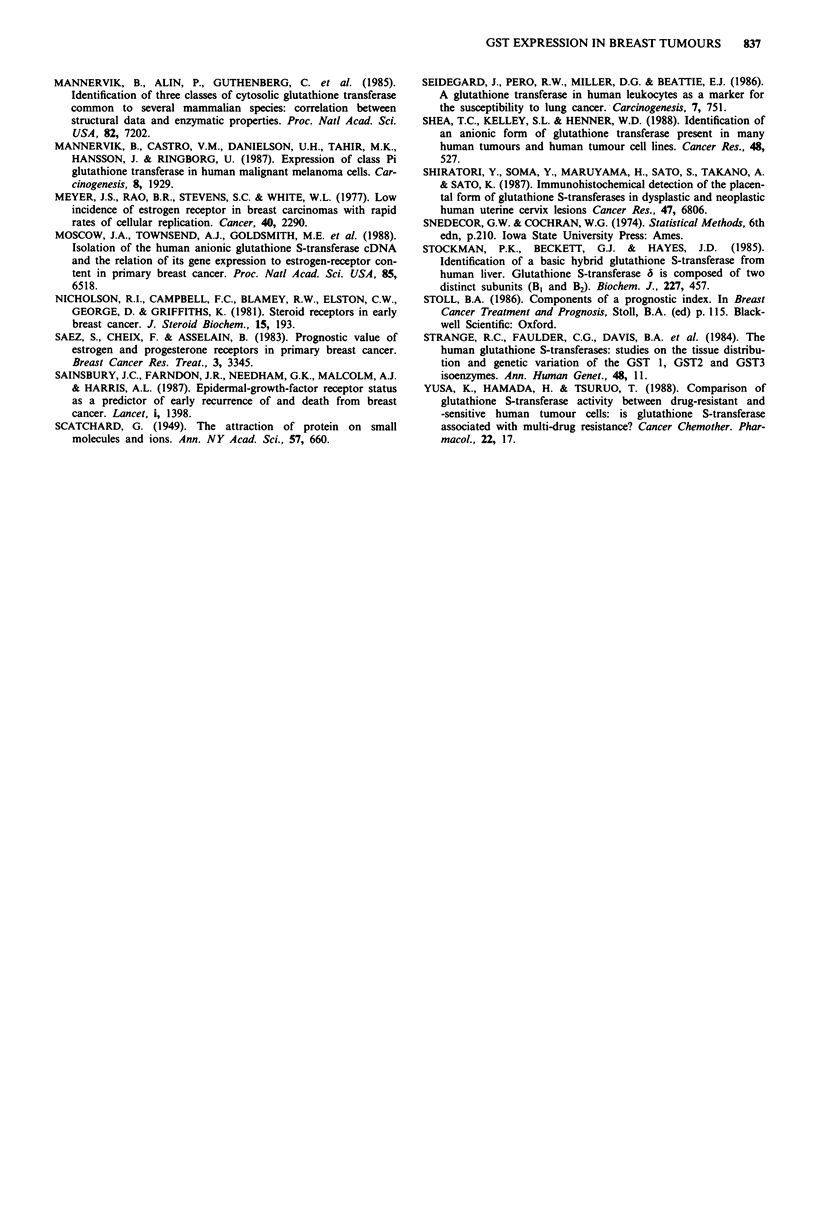


## References

[OCR_00447] Adami H. O., Graffman S., Lindgren A., Sällström J. (1985). Prognostic implication of estrogen receptor content in breast cancer.. Breast Cancer Res Treat.

[OCR_00452] Ali I. U., Lidereau R., Theillet C., Callahan R. (1987). Reduction to homozygosity of genes on chromosome 11 in human breast neoplasia.. Science.

[OCR_00457] Beckett G. J., Hayes J. D. (1984). Development of specific radioimmunoassays for the measurement of human hepatic basic and N/A2b glutathione S-transferases.. Clin Chim Acta.

[OCR_00461] Board P. G., Webb G. C. (1987). Isolation of a cDNA clone and localization of human glutathione S-transferase 2 genes to chromosome band 6p12.. Proc Natl Acad Sci U S A.

[OCR_00465] Bradford M. M. (1976). A rapid and sensitive method for the quantitation of microgram quantities of protein utilizing the principle of protein-dye binding.. Anal Biochem.

[OCR_00470] Buller A. L., Clapper M. L., Tew K. D. (1987). Glutathione S-transferases in nitrogen mustard-resistant and -sensitive cell lines.. Mol Pharmacol.

[OCR_00481] Cattoretti G., Andreola S., Clemente C., D'Amato L., Rilke F. (1988). Vimentin and p53 expression on epidermal growth factor receptor-positive, oestrogen receptor-negative breast carcinomas.. Br J Cancer.

[OCR_00475] Corle D. K., Sears M. E., Olson K. B. (1984). Relationship of quantitative estrogen-receptor level and clinical response to cytotoxic chemotherapy in advanced breast cancer. An extramural analysis.. Cancer.

[OCR_00487] Courdi A., Héry M., Chauvel P., Gioanni J., Namer M., Demard F. (1988). Prognostic value of continuous variables in breast cancer and head and neck cancer. Dependence on the cut-off level.. Br J Cancer.

[OCR_00532] Di Ilio C., Del Boccio G., Massoud R., Federici G. (1986). Glutathione transferase of human breast is closely related to transferase of human placenta and erythrocytes.. Biochem Int.

[OCR_00500] Hawkins R. A., Black R., Steele R. J., Dixon J. M., Forrest A. P. (1981). Oestrogen receptor concentration in primary breast cancer and axillary node metastases.. Breast Cancer Res Treat.

[OCR_00493] Hawkins R. A., Roberts M. M., Forrest A. P. (1980). Oestrogen receptors and breast cancer: current status.. Br J Surg.

[OCR_00504] Hawkins R. A., White G., Bundred N. J., Dixon J. M., Miller W. R., Stewart H. J., Forrest A. P. (1987). Prognostic significance of oestrogen and progestogen receptor activities in breast cancer.. Br J Surg.

[OCR_00515] Howell A., Barnes D. M., Harland R. N., Redford J., Bramwell V. H., Wilkinson M. J., Swindell R., Crowther D., Sellwood R. A. (1984). Steroid-hormone receptors and survival after first relapse in breast cancer.. Lancet.

[OCR_00520] Howie A. F., Hayes J. D., Beckett G. J. (1988). Purification of acidic glutathione S-transferases from human lung, placenta and erythrocyte and the development of a specific radioimmunoassay for their measurement.. Clin Chim Acta.

[OCR_00526] Hussey A. J., Hayes J. D., Beckett G. J. (1987). The polymorphic expression of neutral glutathione S-transferase in human mononuclear leucocytes as measured by specific radioimmunoassay.. Biochem Pharmacol.

[OCR_00538] Jakoby W. B. (1978). The glutathione S-transferases: a group of multifunctional detoxification proteins.. Adv Enzymol Relat Areas Mol Biol.

[OCR_00541] Kodate C., Fukushi A., Narita T., Kudo H., Soma Y., Sato K. (1986). Human placental form of glutathione S-transferase (GST-pi) as a new immunohistochemical marker for human colonic carcinoma.. Jpn J Cancer Res.

[OCR_00546] Leclercq G., Heuson J. C. (1977). Therapeutic significance of sex-steroid hormone receptors in the treatment of breast cancer.. Eur J Cancer.

[OCR_00570] Mannervik B., Alin P., Guthenberg C., Jensson H., Tahir M. K., Warholm M., Jörnvall H. (1985). Identification of three classes of cytosolic glutathione transferase common to several mammalian species: correlation between structural data and enzymatic properties.. Proc Natl Acad Sci U S A.

[OCR_00577] Mannervik B., Castro V. M., Danielson U. H., Tahir M. K., Hansson J., Ringborg U. (1987). Expression of class Pi glutathione transferase in human malignant melanoma cells.. Carcinogenesis.

[OCR_00564] Mannervik B. (1985). The isoenzymes of glutathione transferase.. Adv Enzymol Relat Areas Mol Biol.

[OCR_00583] Meyer J. S., Rao B. R., Stevens S. C., White W. L. (1977). Low incidence of estrogen receptor in breast carcinomas with rapid rates of cellular replication.. Cancer.

[OCR_00588] Moscow J. A., Townsend A. J., Goldsmith M. E., Whang-Peng J., Vickers P. J., Poisson R., Legault-Poisson S., Myers C. E., Cowan K. H. (1988). Isolation of the human anionic glutathione S-transferase cDNA and the relation of its gene expression to estrogen-receptor content in primary breast cancer.. Proc Natl Acad Sci U S A.

[OCR_00595] Nicholson R. I., Campbell F. C., Blamey R. W., Elston C. W., George D., Griffiths K. (1981). Steroid receptors in early breast cancer: value in prognosis.. J Steroid Biochem.

[OCR_00605] Sainsbury J. R., Farndon J. R., Needham G. K., Malcolm A. J., Harris A. L. (1987). Epidermal-growth-factor receptor status as predictor of early recurrence of and death from breast cancer.. Lancet.

[OCR_00615] Seidegård J., Pero R. W., Miller D. G., Beattie E. J. (1986). A glutathione transferase in human leukocytes as a marker for the susceptibility to lung cancer.. Carcinogenesis.

[OCR_00620] Shea T. C., Kelley S. L., Henner W. D. (1988). Identification of an anionic form of glutathione transferase present in many human tumors and human tumor cell lines.. Cancer Res.

[OCR_00626] Shiratori Y., Soma Y., Maruyama H., Sato S., Takano A., Sato K. (1987). Immunohistochemical detection of the placental form of glutathione S-transferase in dysplastic and neoplastic human uterine cervix lesions.. Cancer Res.

[OCR_00636] Stockman P. K., Beckett G. J., Hayes J. D. (1985). Identification of a basic hybrid glutathione S-transferase from human liver. Glutathione S-transferase delta is composed of two distinct subunits (B1 and B2).. Biochem J.

[OCR_00647] Strange R. C., Faulder C. G., Davis B. A., Hume R., Brown J. A., Cotton W., Hopkinson D. A. (1984). The human glutathione S-transferases: studies on the tissue distribution and genetic variation of the GST1, GST2 and GST3 isozymes.. Ann Hum Genet.

[OCR_00653] Yusa K., Hamada H., Tsuruo T. (1988). Comparison of glutathione S-transferase activity between drug-resistant and -sensitive human tumor cells: is glutathione S-transferase associated with multidrug resistance?. Cancer Chemother Pharmacol.

